# Associations between Vitamin B-12 Status and Oxidative Stress and Inflammation in Diabetic Vegetarians and Omnivores

**DOI:** 10.3390/nu8030118

**Published:** 2016-02-26

**Authors:** Yau-Jiunn Lee, Ming-Yang Wang, Mon-Chiou Lin, Ping-Ting Lin

**Affiliations:** 1 Lee’s Endocrinologic Clinic, Pingtung 90000, Taiwan; t3275@ms25.hinet.net; 2 Department of Nutrition, Jen-Ai Hospital, Tali-Taichung 40201, Taiwan; steady9705@gmail.com; 3 Department of Dietetics & Nutrition, Yang Ming Hospital, Chiayi 60045, Taiwan; a0912132648@gmail.com; 4 School of Nutrition, Chung Shan Medical University, Taichung 40201, Taiwan; 5 Department of Nutrition, Chung Shan Medical University Hospital, Taichung 40201, Taiwan

**Keywords:** vitamin B-12, oxidative stress, inflammation, vegetarian, diabetes

## Abstract

Diabetes is considered an oxidative stress and a chronic inflammatory disease. The purpose of this study was to investigate the correlations between vitamin B-12 status and oxidative stress and inflammation in diabetic vegetarians and omnivores. We enrolled 154 patients with type 2 diabetes (54 vegetarians and 100 omnivores). Levels of fasting glucose, glycohemoglobin (HbA1c), lipid profiles, oxidative stress, antioxidant enzymes activity, and inflammatory makers were measured. Diabetic vegetarians with higher levels of vitamin B-12 (>250 pmol/L) had significantly lower levels of fasting glucose, HbA1c and higher antioxidant enzyme activity (catalase) than those with lower levels of vitamin B-12 (≤250 pmol/L). A significant association was found between vitamin B-12 status and fasting glucose (*r* = −0.17, *p* = 0.03), HbA1c (*r* = −0.33, *p* = 0.02), oxidative stress (oxidized low density lipoprotein-cholesterol, *r* = −0.19, *p* = 0.03), and antioxidant enzyme activity (catalase, *r* = 0.28, *p* = 0.01) in the diabetic vegetarians; vitamin B-12 status was significantly correlated with inflammatory markers (interleukin-6, *r* = −0.33, *p* < 0.01) in diabetic omnivores. As a result, we suggest that it is necessary to monitor the levels of vitamin B-12 in patients with diabetes, particularly those adhering to a vegetarian diet.

## 1. Introduction

Data from the Third National Health and Nutrition Examination Survey (NHANES III) indicated that an overall estimated US adult population prevalence of low serum vitamin B-12 status was 3.2%, and the prevalence increased to 4.4% for those aged >50 years [1]. It is well known that a vegetarian diet may influence the vitamin B-12 status because vitamin B-12 is present in most animal food sources, and the low status of vitamin B-12 among vegetarians that results in hyperhomocysteinemia, elevated red blood cell distribution width and mean corpuscular volume predisposes one to circulatory problems, and may negate the health benefits of the vegetarian diet [2]. Metformin is considered a cornerstone in the treatment of diabetes; it not only showed a beneficial effect on reducing levels of plasma glucose but also on lowering lipids profiles [3]. However, data from NHANES 1999–2006 found the prevalence of vitamin B-12 deficiency to be 5.8% in US adults with diabetes under metformin therapy [4] and therefore suggest that long-term treatment of diabetic patients with metformin may cause a higher risk of developing vitamin B-12 deficiency [5].

Diabetes is considered an oxidative stress and a chronic inflammatory disease [6]. Recent studies have indicated that vitamin B-12 is an antioxidant, and a lower status of vitamin B-12 might be a potential trigger contributing to increase oxidative stress, particularly in patients with diabetes [7,8,9]. Vitamin B-12 acts as an antioxidant or anti-inflammation agent, which might modulate oxidative stress responses, including those of inflammatory responses [10,11,12,13,14]. To the best of our knowledge, no clinical study has examined the association between vitamin B-12 status and oxidative stress and inflammation, especially in patients with diabetes who adhere to a vegetarian diet. Therefore, the purpose of this study was to investigate the correlations between vitamin B-12 status and oxidative stress, and inflammation in diabetic vegetarians and omnivores.

## 2. Materials and Methods

### 2.1. Study Design

The current study was a cross-sectional study. We enrolled 154 (64 male and 93 female) adult patients (aged 20–84 years) with type 2 diabetes from Lee’s Endocrinologic Clinic (Pingtung County, Taiwan). The diagnostic criteria for type 2 diabetes were defined as a glycohemoglobin (HbA1c) ≥6.5%, a fasting glucose ≥7.0 mmol/L or a 2-h plasma glucose ≥11 mmol/L during an oral glucose tolerance test (OGTT), as well as the use of anti-hyperglycemic drugs. We excluded patients with liver or renal disease, pregnant women, and using antioxidants or vitamin B-12 supplements. The inclusion criteria for vegetarian subjects were that the subjects consumed no meat or fish, although they were allowed to consume dairy products or eggs, and had maintained a vegetarian diet for at least one year. Fifty-four diabetic vegetarians and 100 omnivores participated in this study. The study was approved by the Institutional Review Board of Chung Shan Medical University Hospital, Taiwan (CSMUH No.: CS12203). Each subject provided written informed consent to participate in the study.

### 2.2. Anthropometric and Dietary Measurements

We measured blood pressures, body weights, heights, and waist and hip circumferences of each patient, and then calculated the body mass index (BMI) and ratios of waist to hip circumference. Blood pressure was measured after each patient rested for at least 5 min. Dietary intake was assessed by dietitians and used 24-h diet recall. The data of nutrients were analyzed using the Nutritionist Professional software package (E-Kitchen Business Corp., Taiwan). The ages, gender, smoking, drinking, exercise habits, and medications of all subjects were recorded.

### 2.3. Blood Collection and Biochemical Measurement

Fasting blood specimens were collected in vacutainer tubes without anticoagulant (Becton Dickinson, Rutherford, NJ, USA). The samples were centrifuged at 3000 rpm for 15 min at 4 °C and the serum was separated. Fasting glucose was measured by Roche Performa glucose meters (Accu-chek, Mannheim, Germany) and glycohemoglobin (HbA1c) was measured by Variant II hemoglobin testing system kits (Bio-Rad Laboratories, Inc., California, CA, USA). Serum total cholesterol (TC), triglyceride (TG), low density lipoprotein-cholesterol (LDL-C), and high density lipoprotein-cholesterol (HDL-C) levels were measured using an automated biochemical analyzer (Hitachi-7180E, Tokyo, Japan). The levels of apolipoprotein A-1 (Apo-A1) and apolipoprotein-B (Apo-B) were measured using polyethylene glycerol (PEG) enhanced immunoturbidimetric assays (Siemens Healthcare Diagnostics Inc., New York, NY, USA).

### 2.4. Serum Vitamin B-12, Oxidative Stress, and Inflammatory Markers Measurements

Serum levels of vitamin B-12 were measured by electrochemiluminescence immunoassay (ECLIA) using a commercially available kit (Cobas^®^Roche, Basel, Switzerland). Serum oxidized LDL-C (Ox-LDL-C) was measured by an enzyme-linked immunosorbent assay (ELISA) using a commercially available kit (Mercodia, Uppsala, Sweden) according to the supplier’s instructions. Plasma malondialdehyde (MDA) was determined using the thiobarbituric acid reactive substances method, as described by Botsoglou *et al.* [15]. Red blood cells (RBCs) were diluted with 25x sodium phosphate buffer for superoxide dismutase (SOD) and glutathione peroxidase (GPx) measurements and 250x sodium phosphate buffer for catalase (CAT) measurement. The methods for measuring CAT, SOD, and GPx in RBCs have been previously described [16,17,18]; these measurements were performed spectrophotometrically at 240 nm, 325 nm, and 340 nm, respectively. Protein contents of RBCs were determined based on the Biuret reaction of the BCA kit (Thermo, Rockford, IL, USA). Antioxidant enzymes activity levels were expressed as unit/mg of protein. All analyses were performed in duplicate and the variations of repeated determinations were within 5% of the same sample. With regard to the inflammatory markers, serum levels of high sensitivity C-reactive protein (hs-CRP) were quantified by particle-enhanced immunonephelometry with an image analyzer (Dade Behring, Deerfield, IL, USA), and serum levels of high sensitivity interleukin-6 (IL-6) were measured by ELISA using a commercially available kit (eBioscience, San Diego, CA, USA).

### 2.5. Statistical Analysis

The data were expressed as the means and standard deviations (SD), as well as the medians. A Kolmogorov-Smirnov test was used to examine the normal distribution of variables. Student’s *t*-test or the Mann-Whitney rank sum test was used to compare mean values for continuous variables between the diabetic vegetarians and omnivores. For the categorical response variables, differences between the two groups were assessed by the Chi-square test or Fisher's exact test. Pearson product moment correlations or Spearman’s rank order correlations were used to examine the correlation between serum vitamin B-12 status (a dummy variable of 1 was set for subjects who had higher levels of vitamin B-12; the variable for lower levels of vitamin B-12 was set at 0) and the levels of blood glucose, oxidative stress, and inflammatory markers. Multiple linear regressions were used to examine the correlations between serum vitamin B-12 status (as an independent variable) and blood glucose, oxidative stress, and inflammatory markers after adjustment for gender and age. The cut-off point of serum vitamin B-12 was set at 250 pmol/L based on the medians of vegetarians and the definition of vitamin B12 deficiency (<150 pmol/L) and borderline deficiency (<200 pmol/L) were according on Pawlak [2]. Statistical significance was set at *p* < 0.05. All statistical analyses were performed using SigmaPlot software (version 12.0, Systat, San Jose, CA, USA).

## 3. Results

### 3.1. Characteristics and Dietary Intake of Subjects

The characteristics and dietary intake of the subjects are shown in Table 1. The means for age (*p* < 0.01) were significantly higher in the diabetic vegetarians than in the omnivores. The diabetic vegetarians had a significantly lower frequency under the metformin (*p* = 0.02) or statin (*p* < 0.01) therapy and lower levels of FG (*p* = 0.06), TC (*p* = 0.07), and HDL-C (*p* = 0.01) than diabetic omnivores. However, the levels of TG (*p* = 0.03), hs-CRP (*p* = 0.01), and IL-6 (*p* = 0.04) were significantly higher in diabetic vegetarians than in omnivores. Regarding the dietary intake, the diabetic vegetarians had significantly lower values of energy (*p* < 0.01), protein (*p* < 0.01), fat (*p* < 0.01), cholesterol (*p* < 0.01), and vitamin B-12 (*p* < 0.01) intake than the omnivores, even energy adjusted.

### 3.2. Levels of Vitamin B-12 in the Diabetic Vegetarians and Omnivores

The levels of vitamin B-12 in the diabetic vegetarians and omnivores are shown in Table 2. The diabetic vegetarians had significantly lower levels of vitamin B-12 than the omnivores (*p* < 0.01) as well as those under metformin (*p* < 0.01) or statin (*p* < 0.01) therapy. There was a significantly higher prevalence of vitamin B-12 deficiency in the diabetic vegetarians than in the omnivores. However, the level of vitamin B-12 was not significantly different between males and females, old and young subjects in both vegetarians and omnivores groups.

### 3.3. Levels of Metabolic Biomarkers after Stratifying by Serum Vitamin B-12

The levels of metabolic biomarkers after stratifying by serum vitamin B-12 are shown in Table 3. With regard to the levels of blood glucose, diabetic vegetarians with higher levels of vitamin B-12 had significantly lower levels of fasting glucose and HbA1c than those with lower levels of vitamin B-12 (fasting glucose, *p* < 0.05; HbA1c, *p* = 0.02) and omnivores (fasting glucose, *p* = 0.01; HbA1c, *p* = 0.04). With regard to lipids profiles, diabetic omnivores with higher levels of vitamin B-12 had significantly higher levels of TC (TC, *p* = 0.02) and LDL-C (LDL-C, *p* = 0.03) than those with lower levels of vitamin B-12. Diabetic omnivores with higher levels of vitamin B-12 had significantly higher levels of HDL-C (*p* = 0.04) than vegetarians.

### 3.4. Levels of Oxidative Stress and Inflammatory Markers after Stratifying by Serum Vitamin B-12

The levels of oxidative stress and inflammatory markers after stratifying by serum vitamin B-12 are shown in Table 4. The diabetic vegetarians with higher levels of vitamin B-12 had significantly higher antioxidant enzyme activity (CAT, *p* < 0.05) than those with lower levels of vitamin B-12. The diabetic omnivores with higher levels of vitamin B-12 had significantly lower oxidative stress (MDA, *p* = 0.06) and inflammation (IL-6, *p* = 0.04) than those with lower levels of vitamin B-12 and the vegetarians (MDA, *p* = 0.01; hs-CRP, *p* = 0.03; IL-6, *p* = 0.02). The diabetic omnivores with higher levels of vitamin B-12 had significantly higher antioxidant enzyme activity (SOD, *p* = 0.04) than the vegetarians.

### 3.5. Correlations between Serum Vitamin B-12 Status and Blood Glucose, Oxidative Stress, and Inflammatory Markers

The correlations between serum vitamin B-12 status and blood glucose, oxidative stress, and inflammatory markers are shown in Table 5. A significant association was found between vitamin B-12 status and fasting glucose (*r* = −0.17, *p* = 0.03), HbA1c (*r* = −0.33, *p* = 0.02), oxidative stress (oxidized low density lipoprotein-cholesterol, *r* = −0.19, *p* = 0.03), and antioxidant enzyme activity (catalase, *r* = 0.28, *p* = 0.01) in the diabetic vegetarians; vitamin B-12 status was significantly correlated with inflammatory markers (interleukin-6, *r* = −0.33, *p* < 0.01) in diabetic omnivores. The similar trends of correlations between vitamin B-12 status and blood glucose, oxidative stress, and inflammatory markers additionally adjustment for gender and age (data not shown).

## 4. Discussion

Mounting evidence has shown that the health benefits of a vegetarian diet in diabetic patients can provide a range of natural products and food forms of benefit for blood glucose and lipid abnormalities in diabetes [19,20,21,22,23]. However, in the present study, we found that diabetic vegetarians had a significantly lower vitamin B-12 status, which might be related to an increase the levels of oxidative stress and inflammation. Vitamin B-12 could potentially be a useful antioxidant, because it can stimulate methionine synthase activity and direct reaction with reactive oxygen and nitrogen species, and through a glutathione sparing effect, can modify signaling molecules to decrease oxidative stress [10,11,12,13,14,24,25]. In addition, vitamin B-12 could also act as an anti-inflammation agent through the mechanisms of down regulation of the transcription factor nuclear factor-kappa B (NF-kB), inhibition of nitric oxide synthase, and promotion of oxidative phosphorylation [25,26,27]. In the present study, we have examined inflammatory and metabolic profiles in patients with vitamin B12 deficiency and without vitamin B12 deficiency, regardless of their dietary habits (data not shown). Diabetic patients with lower vitamin B-12 status had a significantly higher levels of blood glucose (HbA1c, *p* = 0.08) and inflammation (hs-CRP, *p* = 0.03), and significantly lower antioxidant enzymes activity (CAT, *p* = 0.02 and GPx, *p* < 0.01) than those with higher vitamin B-12 status. It appears that if diabetic vegetarians are at a high risk for vitamin B-12 deficiency may increase risk of having lower antioxidant capacity and higher inflammatory status. Thus, we suggest it is necessary to monitor vitamin B-12 status regularly in diabetic vegetarians.

Metformin, the first line drug for treating diabetes, has been reported to potentially decrease vitamin B-12 status [3,4,5]. We observed a lower vitamin B-12 status in the diabetic omnivores under metformin therapy, but that was not reached statistically significant (Table 2), therefore, in this study, we consider a vegetarian diet might be a major cause of vitamin B-12 deficiency. The dietary reference intakes (DRIs) of vitamin B-12 in Taiwan are similar to those of the Institute of Medicine (IOM) in the USA and is 2.4 μg per day. Vegetarians, lack the rich vitamin B-12 source of animal food, and although our vegetarian subjects were included lacto- and ovo-vegetarians, the median intake of vitamin B-12 was still lower (0.2 μg/day, Table 1) than the DRIs. The dietary guideline for vegetarians in Taiwan suggest that vegetarians could consume plant food containing substantial amounts of vitamin B-12, such as edible algae (dried green and purple lavers) to meet the recommendation dietary intake of vitamin B-12. However, algal vitamin B-12 appears to be inactive in humans [27]. Although we did not find a significantly lower vitamin B-12 status in diabetic patients under metformin therapy, there is enough scientific evidence to recommend the supplementation of vitamin B-12 in diabetes who are being treated with metformin, to reduce the risk of developing neuropathy and its consequences [28]. As a result, we support that patients with diabetes, particularly those adhering to a vegetarian diet should intake vitamin B-12 supplements or vitamin B-12 fortified food could maintain adequate vitamin B-12 status and prevent vitamin B-12 deficiency.

Vitamin B-12 plays a dominant role in the utilization of carbohydrates, and a lower vitamin B-12 status may cause hyperglycemia. *In vitro* experiments have indicated that the level of glutathione as well as enzyme activity are lower in vitamin B-12 deficient animals; as a result, vitamin B-12 is beneficial for the regulation of glucose [29]. Some observation studies from India have shown that a lower vitamin B-12 status during pregnancy was associated with higher maternal and off-spring insulin resistance [30,31,32,33,34]. Those results are also supported in our diabetic subjects; we found the levels of fasting glucose and HbA1c were significantly lower in the diabetic vegetarians who had a higher vitamin B-12 status (Table 3), and vitamin B-12 status was significantly negatively correlated with blood glucose, particularly in diabetic vegetarians (Table 5). Because a lower vitamin B-12 status may correlate with impaired glucose tolerance, it is important to monitor vitamin B-12 status in patients with diabetes, particularly those adhering to a vegetarian diet. With regard to the association between vitamin B-12 status and lipid profiles, in an observation study of diabetes subjects from Europe and India, the authors found a negative association between vitamin B-12 and lipid profiles [34]. However, in the present study, we found diabetic omnivores with higher vitamin B-12 status had higher levels of lipid profiles (TC and LDL-C) than those with lower vitamin B-12 status (Table 3); we consider vitamin B-12 status to be positively correlated with lipid profiles, which might be due to animal food containing more fat.

The strength of this study was that it was the first clinical study to investigate the correlation between vitamin B-12 status and oxidative stress, and inflammation in diabetic vegetarians and omnivores. Vitamin B-12 status is significantly correlated with oxidative stress and inflammation in the present study. Because vegetarians are at a higher risk for vitamin B-12 deficiency, that might negate the health benefits of a vegetarian diet for those with diabetes. We suggest that diabetic vegetarians should monitor their vitamin B-12 status regularly and use vitamin B-12 supplements if necessary. Further interventional studies are needed to explore the proper dosage of vitamin B-12 supplements for lower oxidative stress and inflammation in patients with diabetes adhering to a vegetarian diet.

## 5. Conclusions

Vitamin B-12 status is significantly negatively correlated with the levels of blood glucose, oxidative stress (ox-LDL) and positively correlated with antioxidant enzyme activity in diabetic vegetarians; and significantly negatively correlated with the levels of inflammation in diabetic omnivores. We suggest it is necessary to monitor the levels of vitamin B-12 in patients with diabetes, particularly those adhering to a vegetarian diet.

## Figures and Tables

**Table 1:** Basic characteristics and dietary intake of subjects.

	Vegetarians (*n =* 54)	Omnivores (*n =* 100)	*p* Values
females (*n*, %)	38 (70%)	55 (55%)	0.09
age (years)	65.1 ± 11.3 (63.5) ^1^	57.7 ± 10.5 (60.0)	<0.01
duration of diabetes (years)	12.0 ± 8.8 (9.5)	9.4 ± 6.1 (8.0)	0.16
body weight (kg)	62.0 ± 12.6 (59.8)	67.6 ± 14.7 (65.5)	0.05
body mass index (kg/m^2^)	24.9 ± 6.2 (25.5)	26.5 ± 5.7 (25.5)	0.31
waist circumference (cm)	88.0 ± 9.9 (89.3)	88.1 ± 11.7 (86.5)	0.63
hip circumference (cm)	95.1 ± 8.0 (94.5)	97.3 ± 10.3 (96.0)	0.32
waist to hip ratio	0.95 ± 0.15 (0.90)	0.92 ± 0.13 (0.90)	0.45
physical activity ^2^	34 (63%)	67 (67%)	0.23
Metformin therapy (*n*, %)	39 (72%)	89 (89%)	0.02
Statin therapy (*n*, %)	26 (48%)	71 (71%)	<0.01
Metformin + Statin (*n*, %)	21 (39%)	64 (64%)	<0.01
fasting glucose (mmol/L)	7.3 ± 0.9 (6.7)	7.7 ± 2.1 (7.4)	0.06
HbA1c (%)	7.4 ± 1.2 (7.2)	7.7 ± 1.3 (7.6)	0.30
TC (mmol/L)	4.4 ± 0.8 (4.3)	4.6 ± 0.8 (4.6)	0.07
TG (mmol/L)	1.6 ± 1.3 (1.3)	1.4 ± 1.4 (1.1)	0.03
LDL-C (mmol/L)	2.3 ± 0.6 (2.3)	2.4 ± 0.6 (2.3)	0.26
HDL-C (mmol/L)	1.3 ± 1.2 (1.2)	1.4 ± 0.3 (1.4)	0.01
TC/HDL-C	3.6 ± 1.1 (3.4)	3.4 ± 1.0 (3.1)	0.13
Apo-A1 (g/L)	1.3 ± 0.3 (1.3)	1.2 ± 0.3 (1.2)	0.36
Apo-B (g/L)	0.8 ± 0.2 (0.8)	0.8 ± 0.2 (0.8)	0.77
hs-CRP (mg/L)	2.1 ± 2.6 (1.1)	1.5 ± 1.9 (0.8)	0.01
IL-6 (pg/mL)	2.5 ± 1.9 (1.8)	2.0 ± 1.7 (1.5)	0.04
*Dietary intake*			
energy (kcal/day)	1410.0 ± 355.7 (1380.6)	1678.1 ± 478.4 (1621.9)	<0.01
protein (g/day)	45.6 ± 16.7 (43.1)	64.4 ± 23.2 (60.0)	<0.01
protein (g/kcal)	1.98 ± 3.25 (1.57)	1.47 ± 0.17 (1.46)	0.04
% of total calories	12.7% ± 3.1% (12.8%)	15.4% ± 3.5% (14.9%)	<0.01
fat (g/day)	38.0 ± 16.1 (36.5)	58.7 ± 26.3 (55.0)	<0.01
fat (g/kcal)	0.79 ± 1.20 (0.60)	0.89 ± 0.38 (0.86)	<0.01
% of total calories	24.0% ± 8.1% (23.1%)	31.0% ± 10.1% (33.0%)	<0.01
carbohydrate (g/day)	226.1 ± 68.2 (205.7)	225.0 ± 74.0 (217.6)	0.97
carbohydrate (g/kcal)	4.53 ± 6.26 (3.59)	3.43 ± 1.21 (3.37)	0.13
% of total calories	63.3% ± 9.4% (62.1%)	53.9% ± 10.5% (52.9%)	<0.01
cholesterol (mg/day)	50.9 ± 96.0 (1.7)	208.5 ± 158.0 (155.3)	<0.01
cholesterol (mg/kcal)	2.57 ± 13.99 (0.02)	3.19 ± 2.48 (2.42)	<0.01
vitamin B-12 (μg/day)	0.4 ± 0.6 (0.2)	5.5 ± 9.3 (2.9)	<0.01
vitamin B-12 (μg/kcal)	0.02 ± 0.07 (0.003)	0.08 ± 0.14 (0.049)	<0.01

^1^ mean ± SD (medians); ^2^ physical activity: individual exercise at least 3 times every week.

**Table 2:** Serum vitamin B-12 in diabetic vegetarians and omnivores.

	Vegetarians (*n* = 54)	Omnivores (*n* = 100)	*p* Values ^2^
Serum vitamin B-12 (pmol/L)	379.4 ± 333.0 (266.1) ^1^	497.9 ± 292.7 (416.9)	<0.01
Males	(*n =* 16) 342.8 ± 339.3 (265.7)	(*n =* 45) 456.4 ± 294.9 (356.6)	0.02
Females	(*n =* 38) 394.8 ± 333.7 (271.3)	(*n =* 55) 531.9 ± 289.1 (480.1)	<0.01
*p* values ^3^	0.58	0.10	
Age ≥ 65 years	(*n =* 26) 333.4 ± 197.9 (283.4)	(*n =* 20) 469.2 ± 309.1 (362.2)	<0.05
Age < 65 years	(*n =* 28) 340.6 ± 302.9 (235.1)	(*n =* 80) 505.1 ± 290.0 (421.4)	<0.01
*p* values ^3^	0.55	0.42	
Vitamin B-12 deficiency ^4^ (*n*, %)	10 (18.5%)	5 (5.0%)	0.02
Vitamin B-12 borderline deficiency ^4^ (*n*, %)	18 (33.3%)	7 (7.0%)	<0.01
Metformin therapy			
Yes	(*n =* 39) 319.5 ± 194.1 (266.1)	(*n =* 89) 493.2 ± 296.1 (410.0)	<0.01
No	(*n =* 15) 312.9 ± 272.1 (222.0)	(*n =* 11) 535.9 ± 273.6 (480.1)	0.03
*p* values ^5^	0.44	0.55	
Statin therapy			
Yes	(*n =* 26) 358.2 ± 289.6 (251.0)	(*n =* 71) 516.5 ± 303.1 (440.7)	<0.01
No	(*n =* 28) 321.5 ± 249.6 (275.0)	(*n =* 29) 452.4 ± 264.9 (349.0)	0.02
*p* values ^5^	0.54	0.15	
Metformin + Statin therapy			
Yes	(*n =* 21) 331.9 ± 179.5 (252.6)	(*n =* 64) 506.3 ± 307.8 (437.8)	0.01
No	(*n =* 33) 307.9 ± 244.7 (243.1)	(*n =* 36) 483.0 ± 267.3 (367.3)	<0.01
*p* values ^5^	0.26	0.60	

^1^ mean ± SD (medians); ^2^ values were compared between vegetarian and omnivore groups; ^3^ values were compared between gender or age stratification; ^4^ Vitamin B-12 deficiency: serum vitamin B-12 < 150 pmol/L; Vitamin B-12 borderline deficiency: serum vitamin B-12 < 200 pmol/L; ^5^ values were compared between drugs users and non-users.

**Table 3:** Levels of metabolic biomarkers after stratifying by serum vitamin B-12 ^1^.

	Vegetarians (*n =* 54)		Omnivores (*n =* 100)	
	≤250 pmol/L (*n =* 25)	>250 pmol/L (*n =* 29)	≤250 pmol/L (*n =* 15)	>250 pmol/L (*n =* 85)
*Blood glucose*				
fasting glucose (mmol/L)	7.3 ± 1.8 (6.8)	6.5 ± 1.5 (6.3) *^,†^	7.6 ± 1.5 (8.1)	7.1 ± 1.5 (7.1)
HbA1c (%)	7.5 ± 1.1 (7.2)	6.8 ± 0.8 (6.8) *^,†^	7.3 ± 0.8 (7.5)	7.2 ± 0.8 (7.2)
*Lipid profiles*				
TC (mmol/L)	4.2 ± 0.8 (4.1)	4.6 ± 0.9 (4.6)	4.2 ± 0.7 (4.2)	4.7 ± 0.8 (4.7) *
TG (mmol/L)	1.4 ± 0.7 (1.3)	1.8 ± 1.7 (1.3)	1.0 ± 0.4 (1.0)	1.5 ± 1.4 (1.1)
LDL-C (mmol/L)	2.1 ± 0.5 (2.2)	2.4 ± 0.6 (2.3)	2.1 ± 0.6 (2.0)	2.4 ± 0.6 (2.4) *
HDL-C (mmol/L)	1.3 ± 0.4 (1.2)	1.3 ± 0.4 (1.2) ^†^	1.5 ± 0.4 (1.4)	1.4 ± 0.3 (1.4)
TC/HDL-C	3.4 ± 0.8 (3.1)	3.8 ± 1.3 (3.6)	2.9 ± 0.7 (2.6)	3.5 ± 1.0 (3.3)
Apo-A1 (g/L)	1.2 ± 0.3 (1.3)	1.3 ± 0.4 (1.3)	1.2 ± 0.3 (1.2)	1.2 ± 0.3 (1.2)
Apo-B (g/L)	0.8 ± 0.2 (0.8)	0.9 ± 0.3 (0.9)	0.7 ± 0.2 (0.7)	0.8 ± 0.3 (0.8)

^1^ mean ± SD (medians); * values were compared within groups; ^†^ values were compared between vegetarian and omnivore groups at the same stratified level of serum vitamin B-12.

**Table 4:** Levels of oxidative stress and inflammatory markers after stratifying by serum vitamin B-12 ^1^.

	Vegetarians (*n =* 54)		Omnivores (*n =* 100)	
	≤250 pmol/L (*n =* 25)	>250 pmol/L (*n =* 29)	≤250 pmol/L (*n =* 15)	>250 pmol/L (*n =* 85)
*Oxidative stress*				
MDA (μmol/L)	1.6 ± 0.6 (1.3)	1.6 ± 0.3 (1.6)	1.5 ± 0.3 (1.5)	1.4 ± 0.3 (1.4) *^,†^
Ox-LDL-C (U/L)	33.7 ± 8.2 (31.1)	31.0 ± 4.7 (31.7)	31.8 ± 10.9 (28.1)	33.57 ± 7.2 (33.1)
*Antioxidant enzymes*				
CAT (U/mg protein)	19.2 ± 6.8 (19.1)	24.6 ± 10.8 (22.0) *	19.3 ± 5.9 (18.0)	25.5 ± 12.4 (22.4) *
SOD (U/mg protein)	18.4 ± 8.1 (17.0)	14.5 ± 5.7 (14.9)	19.6 ± 7.8 (23.1)	19.6 ± 7.9 (18.6) ^†^
GPx (U/mg protein)	20.0 ± 4.3 (19.5)	20.5 ± 4.9 (20.5)	21.2 ± 4.1 (21.6)	20.1 ± 5.1 (19.8)
*Inflammatory markers*				
hs-CRP (mg/L)	1.5 ± 1.5 (0.9)	2.7 ± 3.2 (1.5)	1.4 ± 1.6 (0.8)	1.2 ± 1.2 (0.8) ^†^
IL-6 (pg/mL)	2.2 ± 1.7 (1.7)	2.7 ± 2.2 (1.8)	2.4 ± 1.7 (2.0)	1.5 ± 0.8 (1.3) *^,†^

^1^ mean ± SD (medians); * values were compared within groups; ^†^ values were compared between vegetarians and omnivores groups at the same stratified level of serum vitamin B-12.

**Table 5:** Correlations between serum vitamin B-12 status ^1^ and blood glucose, oxidative stress, and inflammatory markers.

	Vegetarians (*n =* 54)	Omnivores (*n =* 100)	Pooled (*n =* 154)
	*r* ^2^ (*p* Values)		
*Blood glucose*			
fasting glucose (mmol/L)	−0.17 (0.03)	−0.12 (0.05)	−0.10 (0.03)
HbA1c (%)	−0.33 (0.02)	−0.06 (0.35)	−0.17 (<0.01)
*Oxidative stress*			
MDA (μmol/L)	0.07 (0.63)	−0.11 (0.06)	−0.07 (0.17)
Ox-LDL-C (U/L)	−0.19 (0.03)	0.09 (0.42)	0.00 (0.98)
*Antioxidant enzymes*			
CAT (U/mg protein)	0.28 (0.01)	0.17 (0.03)	0.23 (<0.01)
SOD (U/mg protein)	−0.21 (0.12)	0.00 (0.98)	−0.03 (0.70)
GPx (U/mg protein)	0.06 (0.68)	−0.08 (0.43)	−0.02 (0.78)
*Inflammation*			
hs-CRP (mg/L)	0.19 (0.17)	−0.05 (0.41)	0.03 (0.69)
IL-6 (pg/mL)	0.13 (0.37)	−0.33 (<0.01)	−0.14 (0.02)

^1^ serum vitamin B-12 levels > 250 pmol/L defined as 1; ≤250 pmol/L = 0; ^2^ correlation coefficients.

## Abbreviations

The following abbreviations are used in this manuscript:

HbA1c	glycohemoglobin
Apo	apolipoprotein
CAT	catalase
FG	fasting glucose
GPx	glutathione peroxidase
HDL-C	high density lipoprotein-cholesterol
hs-CRP	high sensitivity C-reactive protein
IL-6	high sensitivity interleukin-6
LDL-C	low density lipoprotein-cholesterol
MDA	malondialdehyde
SOD	superoxide dismutase
TC	total cholesterol
TG	triglycerol
Ox-LDL-C	oxidized low density lipoprotein-cholesterol
